# Editorial: Resilience of grapevine to climate change: from plant physiology to adaptation strategies, volume II

**DOI:** 10.3389/fpls.2023.1268158

**Published:** 2023-08-11

**Authors:** Tommaso Frioni, Chiara Pastore, Maria P. Diago

**Affiliations:** ^1^ Department of Sustainable Crop Production, Università Cattolica del Sacro Cuore, Piacenza, Italy; ^2^ Department of Agricultural and Food Sciences, University of Bologna, Bologna, Italy; ^3^ Department of Agricultural and Food Sciences, University of La Rioja, Logroño, Spain; ^4^ Department of Viticulture, Institute of Grapevine and Wine Sciences, Logroño, Spain

**Keywords:** temperature, berry composition, climate change, *Vitis vinifera* L., soil management, canopy management, breeding

## Introduction

Under a dramatic and progressive increase of vineyard stress incidence, frequency, and unpredictability, new solutions to defend the world wine industry from climate change-related problems are urgently needed. Following Volume I (Pastore et al., 2022), in this Research Topic, 12 studies from 7 different countries provide significant knowledge on the effects of key environmental stressors on specific grapevine physiologic/metabolic traits or propose groundbreaking strategies to improve vineyard resilience to climate change (**Table 1**).

**Table 1:** An overview of the topics and of the adaptation strategies proposed by the 12 papers included in the Research Topic.

Authors	Location	Limiting factors studied						Type of adaptation proposed				
		Water stress	Temperature, humidity and VPD	Light	Salinity	CO_2_	Biotic stresses	Genetic material	Canopy management	Soil management	Irrigation	Foliar applications
Arguedas et al.	CAN		X	X					X			
Oliver et al.	SPA		X						X			
Yu et al.	USA	X	X						X		X	
Ramos et al.	SPA						X	X				X
Daldoul et al.	TUN				X			X				
Dami and Zhang	USA		X					X				X
Wohlfart et al.	GER					X		X				
Costa et al.	POR	X	X							X	X	
Müller et al.	GER	X	X	X					X		X	
Buesa et al.	SPA	X	X	X					X	X		
Fichtl et al.	GER	X						X				
Poni et al.	ITA	X	X	X					X			

## How climate change factors are affecting grapevine morpho-physiology and berry traits

The wine sector faces important challenges related to sustainability issues and the impact of climate change. Extreme climate events, together with the increase of carbon dioxide (CO_2_), have become a matter of concern for the wine sector worldwide. In this context, yield and quality losses due to increasing temperatures, altered solar radiation, and scarce water availability are challenging grape growers, especially if associated with the appearance of sunburn symptoms that can cause sunburn necrosis in berries. Knowledge of the temperature conditions that can induce sunburn on berry surface can be useful to predict the appearance of sunburn damage and to adopt specific mitigation strategies. The probability of a berry developing symptoms of sunburn necrosis depends on both the intensity and duration of the heat that it receives. Müller et al. reported that with longer durations of heat exposure, lower berry surface temperature is required for berries to show symptoms.

Volatile compounds (VCs) play an important role in wine quality and can be considered one of the most important secondary metabolites in the grapevine berry. Volatile compounds biosynthesis in grape berries is affected by environmental conditions and, in particular, by UV-exposure and temperatures of the bunches. Moderate temperature increases and reduced ultraviolet (UV) exposure in different stages of berry ripening (pre-veraison, post-veraison, and ripening) can affect the composition of VCs in *Vitis* sp. ‘L’ Acadie blanc’, in Nova Scotia (Canada), suggesting that climate change events can affect VCs biosynthesis at harvest, even if they occur at early berry developmental stages (Campos-Arguedas et al.). Increasing temperatures can impact the vines directly, as previously described, or through an indirect effect linked to the raising of soil temperature. Soil conditions (topography, properties, and management) influence the vine morpho-physiology, as reviewed by Costa et al. The highest biomass production and shoot growth rates are linked to an increase of soil temperature conditions (24°C compared to 13°C), while the exposure of grapevine roots to high temperatures often decreases primary root length and lateral root density.

The increase of CO_2_ concentration induces increased photosynthesis in most plant species, including *Vitis vinifera* grapevines, but the mechanisms behind this improvement are not completely understood. Elevated CO_2_ can also bring a modification of leaf physiology and morphological leaf characteristics without altering leaf pigments. Wohlfahrt et al. observed that depending on the cultivar, palisade parenchyma could increase and the epidermal tissue could decrease in thickness, suggesting the existence of seasonal adaptation strategies of grapevines under elevated CO_2_ concentrations.

## Plant material to improve climate change resilience

In Volume I, the importance of grapevine genetic diversity in the adaptation to climate change was illustrated. In this second volume, physiological mechanisms and genetic patterns underlying the differential response of varieties and rootstocks to various biotic and abiotic stresses were studied. With regard to better adaptation to increasing drought conditions, the role of rootstocks is essential. Fichtl et al. focused on modeling root architecture traits (e.g., rooting depth, root length density, and specific root length, among others) by integrating interactions with physiological processes, such as different water availability scenarios. In this work, a range of phenotyping methods and the integration of these phenotyping data into different models—to advance the understanding of rootstock x environment x management interactions and to predict rootstock genotype performance in a changing climate—were investigated, and the lessons learnt towards optimizing rootstock breeding were presented. Strongly related to drought increase is the phenomenon of salinity stress. Daldoul et al. revisited the physiological and biochemical response of wild grapevines (*V. Sylvestris*), in particular, that of the genotype “Tababa” versus a well-known rootstock (Paulsen 103), against salinity stress conditions. From this study, the dynamic salt mechanism tolerance of wild grapevines was elucidated, and specific candidate genes that could be valuable for appropriate breeding strategies to increase salt tolerance were identified.

As mentioned, the increase of CO_2_ concentration is another effect of climate change. While elevated CO_2_ induces increased photosynthesis in most plant species, including *Vitis vinifera* grapevines, the mechanisms behind this improvement are not completely understood. Wohlfahrt et al. presented their observations on the leaf physiology and morphological characteristics of cvs. Riesling and Cabernet Sauvignon subjected to elevated CO_2_ conditions using a unique free-air carbon dioxide enrichment set-up (FACE) and provided first insights to seasonal adaptation strategies of grapevines under future elevated CO_2_ concentrations.

## New frontiers on vineyard soil and canopy management

While selecting proper plant material at vineyard establishments will be the foundation of the adaptation of viticulture to climate change, a no lesser role will be taken by technical aspects of seasonal vineyard management, starting from the ground. Indeed, as this Research Topic also confirms, soil management is one of the trending Research Topics. In their extensive review, Costa et al. analyzed vine response at varying soil temperature levels, pointing out that different floor management techniques could affect vine yield and fruit composition by directly modulating water and nutrients availability or weeds competition and also, indirectly, changing soil temperature and organic matter mineralization time evolution. In this framework, the authors acknowledge the relevance of irrigation in order to improve vineyard soil quality and resilience to climate change.

Irrigation was one of the patterns also followed by Müller et al. and Yu et al. In the first article, the authors pointed out that early water deficit could improve grape sunburn tolerance later in the season. Yu et al. showed that different irrigation volumes were interacting with trellis systems at determining vine yield and fruit composition traits, suggesting the revision of an applied water × training system combination in arid environments.

This was also one of the points raised by the review of canopy management by Poni et al. In their article, the authors pointed out the substantial change has occurred in the last twenty years in respect to the directions of such techniques, which were once aimed at boosting ripening and today have the main goal of sheltering clusters from direct exposure or controlling sugars excesses. In this framework, Müller et al. showed that early exposure of grapes to sunlight elicits a priming effect, making grapes more tolerant to sunburn later in the season. Oliver-Manera et al. verified the effects of the vine forcing technique applied after fruit set and at the beginning of bunch closure on yield, vegetative growth, and non-structural carbohydrate reserves over two consecutive years, suggesting that this technique, if allowing the forced vines to restore carbon reserves at the whole-canopy level, could be promising in order to delay harvest. However, in the vineyard climate change adaptation “game”, soil and canopy management cannot be conceived as unrelated topics. Buesa et al. compared canopy shading, bud forcing, and mulching + vine shading *vs* an untreated control, looking for the best strategy to improve cv. Macabeo wines in eastern Spain. The authors found that bud forcing was the most suitable technique for the production of premium sparkling wine, while the additive effects of shading nets and mulching could be the best strategy to improve grapevine physiological performances and grape composition for the production of other styles of wine.

## Foliar applications

With the aim of improving the physiological and biochemical performance of grapevines towards biotic and abiotic stressors, new biological and hormonal products are released to be applied in the leaves. Alternatively, the mechanisms behind the specific response of treated grapevines with one of these products to a particular stressor are sometimes unknown. Two articles covering this approach are included in Volume II. Likewise, the genetic and phenotypic responses of three Mediterranean cultivars to *Dittrichia viscosa* extract and *Bacillus velezensis* strain were investigated under greenhouse conditions (Ramos et al.), showing that markers specific to some differentially expressed genes presented a stable overexpression after being treated (foliar application) with these two biocontrol products, regardless of the grapevine variety. While the global warming of most viticultural areas is predicted as a consequence of climate change, cold damage still remains a strong devastating weather event to grape production in some viticultural regions worldwide. To mitigate freezing stress, grapevines undergo cold acclimation (transition from a cold-sensitive to a cold-hardy state) through a series of physiological changes derived from either the up- or downregulation of more than 3000 genes. As a key player in freezing tolerance, abscisic acid (ABA) and its application at different doses was studied under greenhouse conditions in *Vitis* spp cv. Chamboucin and *Vitis vinifera* cv. Cabernet Franc cultivars (Dami and Zhang). It was revealed that fructose, glucose, and sucrose are the main soluble sugars that correlate with the freezing tolerance of grape buds and that the synthesis of these sugars is enhanced by ABA treatment after raffinose decay in mid-winter.

## Author contributions

TF: Writing – original draft, Writing – review & editing. CP: Writing – original draft, Writing – review & editing. MD: Writing – original draft, Writing – review & editing.
